# Screening of safe soybean cultivars for cadmium contaminated fields

**DOI:** 10.1038/s41598-020-69803-4

**Published:** 2020-07-31

**Authors:** Yang Zhi, Ting Sun, Qixing Zhou, Xue Leng

**Affiliations:** 1 School of Pharmaceutical Engineering, Shenyang Pharmaceutical University, Shenyang, 110016 China; 2 College of Sciences, Northeastern University, Shenyang, 110004 China; 3 Key Laboratory of Pollution Processes and Environmental Criteria (Ministry of Education)/Tianjin Key Laboratory of Environmental Remediation and Pollution Control, College of Environmental Science and Engineering, Nankai University, Tianjin, 300350 China

**Keywords:** Environmental monitoring, Pollution remediation

## Abstract

The selection and breeding of Cd-safe cultivars (CSCs) has been used to minimize the influx of Cd into the human food chain. The pot-culture experiment combined with the field-culture experiment were conducted to screen out CSCs, i.e. the cultivars accumulating Cd at low enough level for safe consumption in their edible parts when grown in contaminated soils, were screened out and explored among the crop cultivars. We used 25 Chinese soybean cultivars in different Cd contaminated soils to assess the performance of this new method. Variations in uptake, enrichment, and translocation of Cd among these cultivars were studied to screen out soybean CSCs. The accumulation of Cd in the five soybean genotypes was lower than 0.20 mg kg^−1^ under 1.0 mg Cd kg^−1^ treatment, and the EF and TF were lower than 1.0. The field studies further identified that cultivar Shennong 10, Tiedou 36 and Liaodou 21 fit the criteria for CSCs, which were suitable to be planted in low-Cd (Cd concentration < 1.22 mg kg^−1^) contaminated soils. The results can provide scientific methods for screening low-Cd accumulation in soybeans and can provide a path for controlling, treating and remedying Cd-contaminated agricultural soils to make grains safe for human consumption.

## Introduction

Metal pollution is a global problem influencing food safety and ecosystem health^1–3^. Cadmium (Cd) inputs to soil via industrial emission, the application of metal-contained sewage sludge, waste disposal and fertilizers, and atmospheric deposition often exceed outputs in crops and drainage waters, thus the content of Cd in many agricultural soils tends to be increased gradually^4–7^. Cadmium pollution in agricultural soil often leads to crop yield reduction^8^, affects the safety of crop and food production^9^, and endangers the sustainability of farmland ecosystem and human health. They are also potential derivatives of cardiovascular disease, reproductive disorders and cancer^10,11^. Soil cadmium levels in China, France and some other countries have been reported to exceed 100 mg/kg^12,13^. In China, cadmium poses a serious threat to the safety of crops and food production, with at least 13,330 hectares of farmland contaminated to varying degrees by cadmium in more than 11 provinces. About 314,750 hectares of farmland in Japan are reported to be contaminated with cadmium^14^. In a word, cadmium pollution in soil, crops and food has become a potential agricultural and environmental problem worldwide^15^. In order to reduce potential human health risks, it is necessary to limit the concentration of cadmium in crops for human consumption^16^.

Fortunately, more and more attention has been paid to the effects of toxic metals in contaminated soil on the growth and quality of crops. There are physical, chemical and biological methods for remediation of heavy metal contaminated soil, but these methods are insufficient in effectiveness, sustainability and economy^17^. In recent years, the production of low Cd crop varieties can be used as a means to reduce the risk of Cd migration into human diet^16^. To screen and explore cadmium safe varieties from crop varieties, that is, varieties with low cadmium content in edible parts grown in polluted soil and safe to eat^18,19^. The concept of CSCs is grounded on the basis of prior studies, which have shown that the uptake and accumulation of metal pollutants by plants not only differ among species, but also among cultivars^20^. Genotypic variation in Cd accumulation has been investigated in rice (*Oryza sativa* L.)^13,21,22^, wheat (*Triticum aestivum* L.)^23,24^, maize (*Zea mays* L.)^25^, barley (*Hordeum Vulgare* L.)^5,26^, and potato (*Solanum tuberosum* L.) ^27,28^. Furthermore, the strategy of selecting CSCs has been proposed for crops and applied successfully to sunflower and durum wheat^29–31^. However, there is limited information about the screening out of CSCs among Chinese soybean cultivars.

Soybean (*Glycine max* L.) is one of the most important food crops in the world. It has been known for more than 5,000 years. Soybean is rich in nutrition, can reduce blood pressure, enhance human immunity, and promote bone growth. Because of the high economic benefits and suitable natural conditions for planting soybean, farmers are more willing to grow soybean in the world. The amount of Cd that enters human diets from a crop depends on the amount of Cd accumulated in the parts that are consumed, so the translocation of Cd within soybean plants, especially into its seed, is very important for human Cd intake through human diets. Thus, decreasing the content of Cd in soybean seeds is extremely important. The results of this work can provide scientific methods for screening low-Cd accumulation in soybeans and they can provide a path for controlling, treating and remedying Cd-contaminated agricultural soils to make crops safety for human consumption.

## Results

### Tolerance of 25 soybean cultivars by Cd stress in the pot-culture experiment

**Figure 1:**
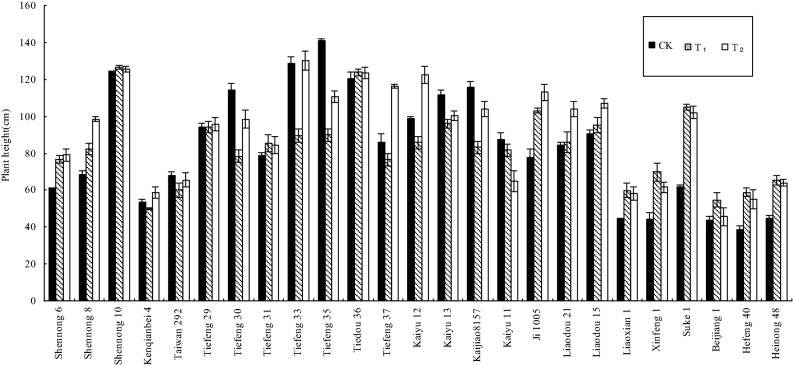
The height of various soybean cultivars in the pot-culture experiment.

In Fig. 1, it describes the plant height of 25 soybean cultivars in different Cd treatments. The plant height of Taiwan 292, Tiefeng 33 and Tiedou 36 under various Cd treatments did not significantly (P > 0.05) differ from CK. In particular, there was a significant (P < 0.05) increase in the plant height of the 17 cultivars (Table 3) under treatments (T_1_ and T_2_) as compared to CK. The result indicated that the above-mentioned Chinese soybeans had tolerance to Cd toxicity. However, the plant height of Tiefeng 30, Tiefeng 35, Kaiyu 13, Kaiyu 11 and Kaijiao 8,157 under T_1_ and T_2_ decreased significantly (P < 0.05) when compared with CK. It indicated that the five soybean cultivars had poor tolerance to Cd stress. Meanwhile, all the tested cultivars grew normally under Cd stress and had no obvious toxicity symptoms between various treatments.

**Figure 2:**
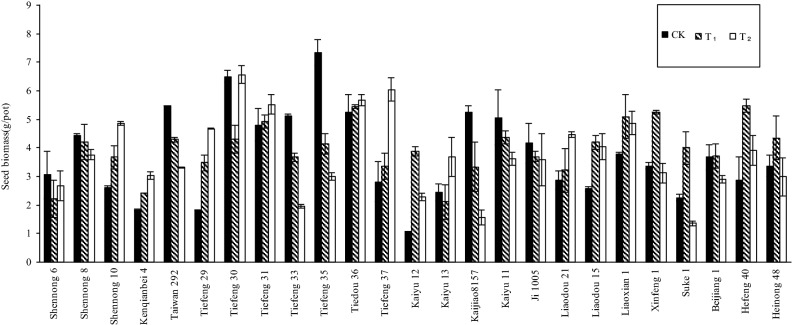
The seed biomass of various soybean cultivars in the pot-culture experiment.

The shoot biomass of soybean plants can also be used to assess plant tolerance to Cd. The seed biomass of 25 cultivars under T_1_ and T_2_ differed significantly (P < 0.05) from that in the control (Fig. 2). The interactions between Cd treatments and soybean cultivars were highly significant (P < 0.05) for seed weight, indicating that the effects of Cd on the growth and development of various soybean cultivars differed obviously. By their changes in seed weight under T_1_ and T_2_, the 25 soybean cultivars can be divided into four groups: (1) the seed weight decreased significantly with an increase in the Cd concentration of Cd in soil, such as Shennong 8, Taiwan 292, Tiefeng 33, Tiefeng 35, Kaijiao 8,157, Kaiyu 11, and Ji 1,005, indicating their poor tolerance to Cd; (2) the seed weight firstly decreased under the 1.0 mg Cd kg^−1^ treatment and then increased under the 2.5 mg Cd kg^−1^ treatment, such as Shennong 6, Tiefeng 30, and Kaiyu 13; (3) the seed weight firstly increased under T_1_ and then decreased under T_2_, such as Kaiyu 12, Liaodou 15, Liaoxian 1, Xifeng 1, Suke 1, Beijiang 1, Hefeng 40, and Heinong 48; and (4) the seed weight increased significantly with an increase in the concentration of Cd in soil, such as Shennong 10, Kenqianbei 4, Tiefeng 29, Tiefeng 31, Tiedou 36, Tiefeng 37 and Liaodou 21, indicating their higher tolerance to Cd toxicity.

### Cd accumulation and distribution in 25 soybean cultivars in the pot-culture experiment

The concentration of Cd in seeds of 25 soybean cultivars was listed in Table 1. It showed that there was a significant (P < 0.05) difference in Cd accumulation among 25 cultivar seeds under T_1_ and T_2_, ranging from 0.09 to 0.98 and from 0.29 to 2.06, and with the mean Cd accumulation of 0.44 and 0.81 mg kg^−1^ DW (dry weight) respectively. It demonstrated that Cd concentrations of 80% (20/25) of the samples examined under T_1_ exceeded the MPC, and 36% were especially higher than 0.50 mg kg^−1^ DW. Because soybean seeds are the edible parts for human beings, the concentration of Cd in soybean seeds should be lower than 0.20 mg kg^−1^ DW and regarded as a rigorous standard for screening out CSCs among 25 soybean cultivars. According to the accumulation of Cd in seeds, the 5 soybean cultivars including Shennong 10, Tiefeng 31, Tiedou 36, Tiefeng 37 and Liaodou 21 were selected as potential Cd excluders or low Cd accumulators under T_1_. However, the Cd concentration in soybean seeds under T_2_ all exceeded 0.20 mg kg^−1^. The highest Cd concentration under T_2_ was found in Hefeng 40 that had great ability to absorb Cd. Meanwhile, the highest Cd concentration in the seeds was tenfold and sevenfold higher than the lowest Cd concentration in the seeds under 1.0 and 2.5 mg Cd kg^−1^ treatments, respectively.

**Table 1:** The Cd concentration in the seeds of 25 Chinese soybean cultivars under different Cd treatments in the pot-culture experiment (mg kg^−1^ DW) (detection limits = 0.01 mg kg^−1^).

Cultivars	Control	1.0 mg kg^−1^	2.5 mg kg^−1^
Shennong 6	0.19 ± 0.13^a^	0.58 ± 0.13	0.94 ± 0.10
Shennong 8	0.07 ± 0.01	0.33 ± 0.03	0.80 ± 0.10
Shennong 10	0.11 ± 0.05	0.14 ± 0.03	0.29 ± 0.03
Kenqianbei 4	0.17 ± 0.15	0.49 ± 0.01	1.38 ± 0.03
Taiwan 292	0.16 ± 0.02	0.39 ± 0.09	0.58 ± 0.03
Tiefeng 29	0.20 ± 0.01	0.59 ± 0.05	0.70 ± 0.05
Tiefeng 30	0.15 ± 0.03	0.93 ± 0.01	1.21 ± 0.06
Tiefeng 31	ND	0.09 ± 0.11	0.51 ± 0.07
Tiefeng 33	0.16 ± 0.01	0.48 ± 0.18	0.97 ± 0.27
Tiefeng 35	0.10 ± 0.04	0.28 ± 0.02	1.02 ± 0.02
Tiedou 36	0.08 ± 0.01	0.18 ± 0.01	0.88 ± 0.04
Tiefeng 37	0.08 ± 0.01	0.19 ± 0.21	1.13 ± 0.04
Kaiyu 11	0.19 ± 0.09	0.77 ± 0.11	0.99 ± 0.06
Kaiyu 12	0.09 ± 0.01	0.39 ± 0.08	1.28 ± 0.07
Kaiyu 13	0.11 ± 0.02	0.42 ± 0.01	0.55 ± 0.01
Kaijiao 8,157	0.18 ± 0.01	0.39 ± 0.01	0.59 ± 0.02
Ji 1,005	0.07 ± 0.01	0.29 ± 0.05	0.51 ± 0.02
Liaodou 21	ND	0.19 ± 0.01	0.65 ± 0.23
Liaodou 15	0.16 ± 0.01	0.58 ± 0.01	0.62 ± 0.08
Liaoxian 1	0.18 ± 0.05	0.62 ± 0.02	0.85 ± 0.06
Xinfeng 1	0.19 ± 0.17	0.51 ± 0.15	1.11 ± 0.12
Suke 1	0.13 ± 0.07	0.29 ± 0.04	0.44 ± 0.04
Beijiang 1	ND	0.46 ± 0.03	1.10 ± 0.20
Hefeng 40	0.18 ± 0.10	0.98 ± 0.08	2.06 ± 0.49
Heinong 48	0.17 ± 0.02	0.73 ± 0.09	1.19 ± 0.33

*ND* not detected.

^a^Means ± S.D. (n = 3).

**Figure 3:**
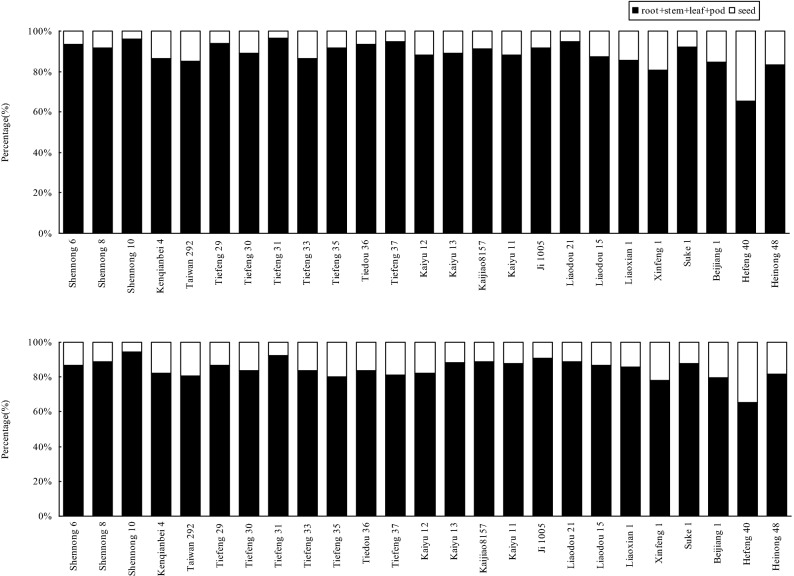
Distribution of Cd in the tissues of various soybean cultivars under two Cd treatments in the pot-culture experiment.

There was no significant (P > 0.05) difference in distribution of Cd in the tissue of the tested cultivars under two treatments except Hefeng 40 (Fig. 3). Less than 20% of Cd in the selected soybean cultivars distributed in the seeds under T_1_ except Xinfeng 1 and Hefeng 40. It indicated that only a small part of Cd could be distributed to the edible portions. And 35% of the total Cd uptake was distributed in the seed of Hefeng 40 under T_1_. However, under T_2_, more Cd was accumulated in the seed compared with that under T_1_. The phenomenon can indicate that the accumulation of Cd in seed will increase to some extent with increasing Cd concentration in soils. While no more than 30% of the total Cd uptake was distributed in the seed of the selected cultivars under T_2_ except Hefeng 40. Thus, the absolute majority of Cd absorbed by soybean plants was retrained in roots, stems, leaves and pods, and only a very small portion of the Cd accumulation was transferred into soybean seeds.

### Enrichment factor and translocation factor of 25 soybean cultivars in the pot-culture experiment

**Figure 4:**
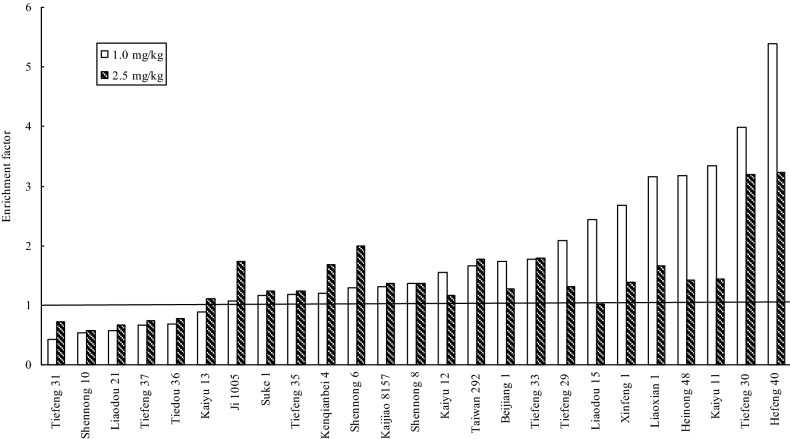
Average enrichment factor of 25 Chinese soybean cultivars under two Cd treatments.

It is a significant (P < 0.05) difference among different soybean cultivars under T_1_ and T_2_, ranging from 0.42 to 5.40 and from 0.24 to 3.23, and with the mean of 1.81 and 1.43 respectively (Fig. 4). The EF value in 72% (18/25) of the tested cultivars decreased with an increase in the concentration of Cd in soils. The highest EF value under the two treatments was found in Hefeng 40, up to 5.40 and 3.23 respectively. Under the two Cd treatments, the EF value in the 5 soybean genotypes including Shennong 10, Tiefeng 31, Tiedou 36, Tiefeng 37 and Liaodou 21 was lower than 1.0. In other words, the 5 genotypes had poor ability to uptake Cd from soils.

**Figure 5:**
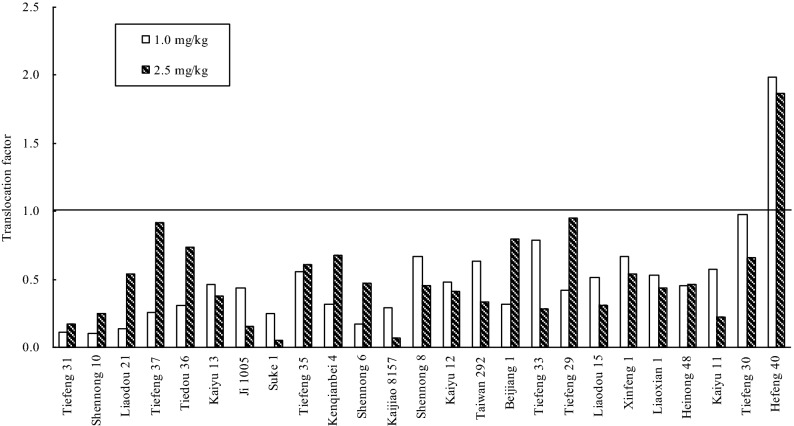
Average translocation factor of 25 Chinese soybean cultivars under two Cd treatments.

The average TF value of different soybeans under T_1_ and T_2_ was from 0.09 to 1.99 and from 0.05 to 1.87 respectively (Fig. 5). Under the Cd treatments, the TF value was all lower than 1.0, excepting Hefeng 40. Being similar with the EF value, the highest TF value was also found in Hefeng 40, up to 1.99 and 1.87 respectively. It indicated that Cd uptake in soybean cultivars was limited in their roots, and only a small portion of Cd could be translocated to the edible parts except Hefeng 40. In a word, the EF and TF values in Shennong 10, Tiefeng 31, Tiedou 36, Tiefeng 37 and Liaodou 21 were lower than 1.0.

### Accumulation and translocation of Cd in 25 soybean cultivars in the field-culture experiment

**Figure 6:**
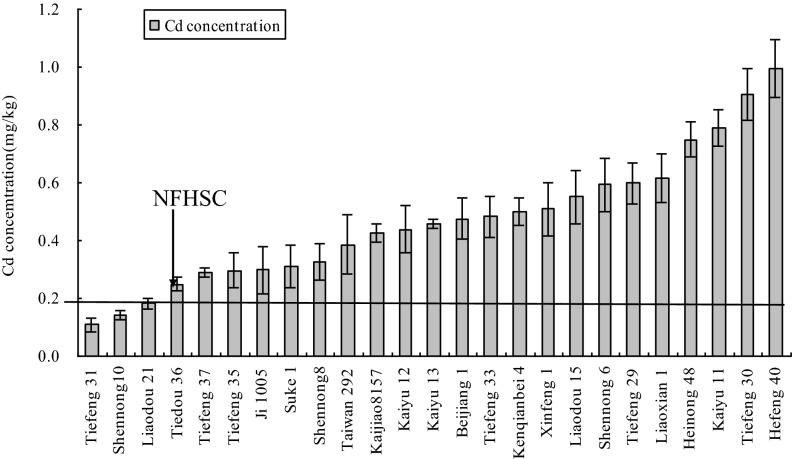
Cd concentration in 25 Chinese soybean cultivars in the field-culture experiment.

**Figure 7:**
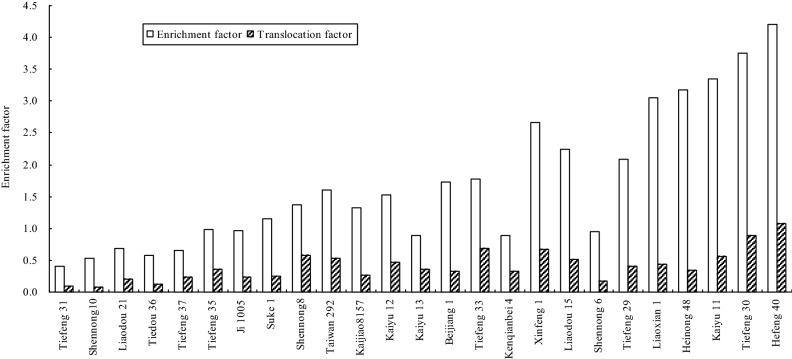
Translocation factor and enrichment factor in 25 Chinese soybean cultivars in the field-culture experiment.

The twenty-five soybean cultivars were further tested in the field-culture experiment to confirm the consistency of genotypic in soybean cultivars between the closed systems and the open systems. In the field-culture experiment, the growth and the yield of 25 soybean cultivars were not limited compared with that under T_1_ in the pot-culture experiment. The Cd concentrations, EF and TF were shown in (Figs. 6 and 7). The Cd concentrations in the 25 soybean cultivars ranged from 0.11 to 0.99 mg kg^−1^, with a mean of 0.47 mg kg^−1^. The Cd concentrations in Tiefeng 31, Shennong 10 and Tiedou 36 were lower than 0.20 mg kg^−1^ (MPC), which was similar to the results under T_1_ in the pot-culture experiment. However, the Cd concentrations in other 22 soybean cultivars were all higher than 0.20 mg kg^−1^. The EF and TF of different soybean cultivars in the field-culture experiment was from 0.41 to 4.2 and from 0.08 to 1.07, respectively, which was relatively lower than that in the pot-culture experiment. The highest Cd concentrations, EF and TF were found in Hefeng 40 in the field-culture experiment, which was similar to the results in the pot-culture experiment. The EF and TF in Tiefeng 31, Shennong 10 and Tiedou 36 were all lower than 1.0.

### Correlation of seed Cd concentrations between the pot-culture and field-culture experiments

**Figure 8:**
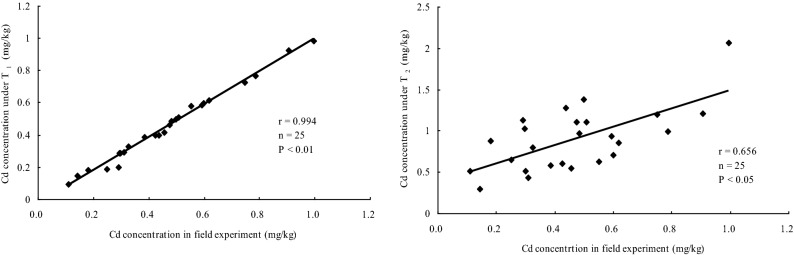
Correlation of Cd concentrations in seed between the pot and the field experiment.

Correlation of seed Cd concentrations between the field-culture experiment and the pot-culture experiment under T_1_ was significantly positive(r = 0.994, n = 25, P < 0.01) (Fig. 8). Meanwhile, correlation of seed Cd concentrations was also found between the field-culture experiment and the pot-culture experiment under T_2_ (r = 0.656, n = 25, P < 0.05).

## Discussion

In recent years, the pollution of Cd in agricultural soil has attracted worldwide attention^32,33^, mainly because of its strong toxicity to human health. Therefore, breeding crop varieties with high Cd resistance or high tolerance, which can improve crop yield potential and reduce the accumulation of heavy metals in edible parts, is of great significance for safe crop and food production.

In the pot experiment, the results showed that not only four fifths of the 25 tested soybean varieties had no significant decrease in plant height (Fig. 1), However, 72% (18/25) of the tested cultivars showed no significant decrease in seed biomass in soils treated with two Cd treatments (Fig. 2), and even 7 cultivars showed significant increase in seed biomass in soils treated with two Cd treatments. The results showed that the tested soybean had a certain degree of tolerance to soil Cd toxicity. Similar positive and neutral responses of biomass to heavy metal stress were also observed in crops such as asparagus bean^19^, rice^34^, Chinese cabbage^35^ and tomato^36^. If the farmer looks at the crop from the outside, (such as biomass and height), they may not get enough warning about the uptake of Cd by crops in time. Therefore, the breeding of CEGs will directly and effectively reduce the potential risk of heavy metals entering the human food chain. However, in the previous literature, there are few discussions on the positive and neutral responses of plant growth under low cadmium levels. Two possible reasons can be suggested. One possible reason is that metal ions can act as activators of enzymes involved in cytokinin metabolism and promote plant growth^37^. Another possible reason is that low concentration of cadmium hyperpolarizes the root surface membrane and increases the transmembrane potential, which is the energy source of cation absorption^38^. Therefore, the positive and neutral responses of plant growth under heavy metal stress need further study.

In recent years, the screening and cultivation of CSCs has attracted wide attention. Zhu et al. found that in Cd contaminated soil (Cd concentration was 0.8 and 1.2 mg kg^−1^, respectively), the average Cd content in fruits of all tested asparagus bean varieties was 0.012 and 0.011 mg kg^−1^, respectively^19^. Chen et al. reported that Cd content in barley grains ranged from 0 to (not detected) to 1.21 mg kg^−1^ DW. Of the 600 barley varieties, nearly half (283/600) of the seed samples exceeded the maximum permissible concentration (MPC) for Cd, with 6.7% of the samples exceeding 0.5 mg kg^−1^ DW, although 0.15 mg kg^−1^ DW was present in the soil^5^. Xin et al. reported that the stability of Cd and Pb accumulation in the shoots of Cd and Pb safe varieties was studied in this experiment. The migration potential of Cd and Pb in water spinach (*Ipomoea aquatica Forsk*). Soils with different Cd and Pb contents was studied^39^. Liu et al. found that there were significant (P < 0.05) differences in Pb content in the shoots of 30 Chinese cabbage cultivars under 500 and 1,500 mg kg^−1^ treatments, ranging from 0.52 to 8.68 and 1.86 to 16.2, with mean values of 3.01 and 6.87 mg kg^−1^DW, respectively^35^. Similar results were observed in this study (Table 3). The seed Cd concentrations of 25 soybean varieties ranged from 0.09 to 0.98 mg kg^−1^ DW under 1.0 mg Cd kg^−1^ treatment, and 80% of the tested soybean varieties had seed Cd concentrations over 0.2 mg kg^−1^, which exceeded the MPC of Cd in soybean. This means that even in low cadmium polluted soil, the concentration of Cd in some soybean seeds exceeds the concentration harmful to human body. Therefore, it is necessary to breed soybean varieties with low Cd accumulation. Especially in China, the most populous country in the world, soil Cd pollution has become one of the important obstacles to sustainable agricultural development^5,14^.

Up to now, there is no clear screening criteria for CSCs. Based on previous studies^40,41^, this study used four criteria for screening food safety CSCs: (1) The concentration of Cd in the edible fraction should be lower than that of MPC (0.2 mg kg^−1^ DW); (2) EF < 1.0; (3) TF < 1.0; (4) As measured by aboveground biomass and height, they can tolerate Cd in polluted soil. In the pot-culture experiment, when the Cd concentration (< 0.2 mg kg^−1^) using edible portion as the standard, five soybean varieties including Shennong 10, Tiefeng 31 and Tiedou 36. Tiefeng 37 and Liaodou 21 in Table 3 could be selected as CSCs under T1 treatment (1.0 mg kg^−1^). In addition, EF and TF values were lower than 1.0 (Figs. 4 and 5). Shennong 10, Tiefeng 31, Tiedou 36, Tiefeng 37 and Liaodou 21 were also used as CSCs (Fig. 2 and Table 2).In the field cultivation experiment, only Tiefeng No. 31 was used, and Shennong 10 and Tiedou 36 were finally determined as CSCs (Fig. 6). However, under treatment T2 the Cd content in the edible part of 25 soybean cultivars was more than 0.2 mg kg^−1^ DW. In other words, the Cd accumulation in the edible part of 25 soybean cultivars could not be considered as CSCs under T2 treatment with higher Cd content in soil. Therefore, the selected CSCs are only suitable for planting soybean in contaminated soil with low Cd content. In this study, Tiefeng 31, Shennong 10 and Liaodou 21 can be confirmed to be CSCs. In addition, Cd concentration, EFs and TFs of Hefeng 40 were the highest in pot and field experiments, which may pose a high risk to human and animal health through the food chain.

**Table 2:** Basic physicochemical property and total Cd concentration in the tested soil.

Soil property	Pot-culture soil	Field-culture soil
pH	6.50	5.50
CEC (cmol kg^−1^)	23.7	22.8
Clay (%)	22.1	18.4
Silt (%)	43.4	43.8
Sand (%)	34.5	37.8
Total-N (%)	0.89	0.97
Total-P (mg kg^−1^)	10.35	10.43
Total-K (mg kg^−1^)	10.96	12.57
TOC (%)	1.52	1.75
Total Cd (mg kg^−1^)	0.15	1.22

A systematic screening method was used to study the effects of endogenous and environmental factors and their interactions on Cd uptake in soybean cultivars by pot and field screening. Kurz et al.'s pot and field experiments on thallium uptake by different rapeseed cultivars showed the same results^42^. However, Liu et al. found that Hengyu 80 was confirm as a pollution-safe variety (PSCs) by treatment of 500 mg Pb kg^−1^ in soil under pot condition, but it could not be considered as PSCs due to that high Pb (> 2.0 mg kg^−1^) in its branches in the field cultivation experiment^35^. In this study, Tiedou36 and Tiefeng37 were also identified as CSCs in T1 culture. However, in the field culture experiments, because the Cd concentration of seeds is higher than that of MPC, they cannot be considered as CSCs. Obviously, rhizosphere conditions in a closed system are different from those in an open system. In addition, there was a significant positive correlation between seed Cd concentration in field culture experiment and that under low level Cd stress. But not the high level of Cd stress in the pot experiment (Fig. 8). The results showed that the effect of environmental factors on Cd uptake was much greater than that of internal factors.

Previous studies have shown that Cd can affect the absorption of other nutrients by crops. Even affect the quality of crops. Liu et al. found that Cd had the most significant effect on the contents of mineral elements in rice roots and leaves, but the results were different for different metal elements, different plant organs and different growth stages^17^. Chen et al. showed that there was a positive correlation between Cd and Zn accumulation. For Cu and Fe accumulation in barley grains, only Mn accumulation had a synergistic effect on Cd accumulation^5^. Liu et al. reported that Cd inhibited the accumulation of K, P, Ca, Mg, Mn and Zn in the aerial part of Chinese cabbage, and the degree of inhibition was different in different varieties^41^. Different reports also give different results about the effect of cadmium on the absorption of microelement^43–45^. Therefore, Cd pollution can cause changes in the uptake and transport of other nutrients by crops, which is a very complex problem. However, there are few reports about the correlation between Cd and nutritional elements of soybean. Therefore, the relationship between Cd and other nutrient elements in Chinese soybean should be further studied.

Heavy metal pollution rarely occurs on a single metal^18^. For example, Chinese cabbage and cucumber produced in about 10,000 hm^2^ of farmland in Shenyang suburb were found to be polluted by Pb and Cd in Liaoning Province, China, and the pollution of Pb, Cd and Hg respectively^46^. Chao et al. reported that much of the land around the Shenyang smelter in northeastern China was found to be heavily contaminated with Cd, Pb and Zn^47^. Bahemuka and Mubov found that vegetables grown along the Sinza and Msimbazi rivers in Dares Salaam, Tanzania, Dares Salaam had relatively high levels of Cd, Pb and Zn in Tanzania. Therefore, our next step is to select CSCs in the case of multi-metal pollution, and verify the feasibility of CSCs strategy^48^.

In this study, clear criteria for screening CSCs were explored. Therefore, only in the polluted soil with low Cd content, the CSCs strategy of soybean varieties is reasonable and feasible. This study provided a scientific basis for selecting soybean varieties with low Cd accumulation, and could be used as a long-term effective and economic means to reduce Cd pollution in crops. Although variety selection will be effective and easy for consumers to improve safer crop and food production, at present, there are still some constraints in the selection process of CSCs. The content of heavy metals in plant tissues varies with the degree of soil pollution, plant genotype and environmental factors^49^. Therefore, further studies to understand the CSCs genotype and environmental effects and their interaction mechanism, as well as the mechanism of pollutant absorption, migration and accumulation in CSCs, is a new direction for the establishment and cultivation of CSCs.

## Conclusions

A systematic screening methodology, pot-culture combined with field-culture screening, was used to identify CSCs in this study. In the pot-culture experiment, the Cd accumulation in seeds of 25 soybean genotypes had significant (P < 0.05) differences under T_1_ and T_2_, with the mean of 0.44 and 0.81 mg kg^−1^ DW, respectively. The accumulation of Cd in the five soybean genotypes was lower than 0.20 mg kg^−1^ under T_1_, and the EF and TF were lower than 1.0. There were significant positive correlation of seed Cd between the field-culture experiment and the pot-culture experiment. Therefore, Shennong 10, Tiedou 36 and Liaodou 21 can be regarded as CSCs, which were safe for consumers only when they were cultivated in the low Cd-contaminated soils( Cd concentration should be lower than 1.22 mg kg^−1^).

## Methods

### Experimental site and soil characterization

A pot-culture experiment was carried out under the open field conditions in the Shenyang Station of Experimental Ecology, Chinese Academy of Sciences (41° 31′ N and 123° 41′ E), which is located at the south of Shenyang City, Liaoning Province, China. Top meadow burozem soil (0–20 cm) was collected from an agricultural area in the station. The average annual temperature in this site was about 6–10 °C and the precipitation was 650–700 mm. The frostless duration was 127–164 days per year. Meanwhile, a field-culture experiment was carried out on a farm (41° 41′ N and 123° 55′ E) in the Dongling District, Shenyang City, Liaoning Province, China. The site meteorology is basically similar to the pot culture experiment site. The basic physical and chemical properties of the pot and field soils were analyzed by the routine analytical methods for agricultural chemicals in soils^50^. In Table 2 it showed the details of the basic properties in soils.

### Experimental design

The soil samples were ground to pass through a 4 mm sieve, after mixing with appropriate amount of Cd (in CdCl_2_·2.5H_2_O solution), then 2.5 kg of soil samples were filled into each plastic pot (Φ = 20 cm, H = 15 cm). There were three treatments including CK (the control, without Cd spiked to soil) and two Cd treatments including T_1_ (1.0 mg Cd kg^−1^ soil) and T_2_ (2.5 mg Cd kg^−1^soil) were applied. The two levels (1.0 and 2.5 mg Cd kg^−1^ soil) stood for low and serious contaminations, according to the evaluation methods of single pollution index and the grading standard of polluted soils by heavy metals^18,35,51^. The soil was watered and then left to equilibrate completely outdoors under a waterproof tarpaulin for about 4 weeks. The period is long enough for natural equilibration of the various sorption mechanisms in the soil. These pots were arranged in randomized complete square design with treble replicate in order to minimize experimental errors.

Soybean seeds were obtained from a seed company in Shenyang, China. Twenty-five soybean cultivars of different origins were used in the pot-culture experiment and the field-culture experiment (Table 3). They were sterilized in 2% (v/v) hydrogen peroxide for 10 min and then washed several times with distilled water. Then six seeds per pot were sowed directly into the soil in the pots. In order to imitate field conditions, soybeans in pots were allowed to grow under the open field conditions and no fertilizers were applied. Using tap water (no Cd detected) to maintain 75% of the field water-holding capacity, and a dish was placed under each pot to gather latent leachate during the experiment period. Within 2 weeks after germination, the seedlings were thinned to three strains in per pot. The selected seedlings were about 7 cm in height with 2 leaves. All tested soybeans were harvested at the seed-maturity stage.

**Table 3:** Different cultivar origin of 25 Chinese soybean cultivars.

Cultivar	Cultivar origin	Growing time (d)
	Female parent × male parent	
Shennong 6	Fengjiao66-12 × Kaiyu 8	137
Shennong 8	Shennong92-16 × Tiefeng29	136
Shennong 10	Shennong92-16 × Shennong91-44	129
Kenqianbei 4	Bei93454 × Heihe18	114
Taiwan 292	–	85–95
Tiefeng 29	Tie8114-7-4 × Tie84059-13-8	130–133
Tiefeng 30	Tiefeng25 × Tie84018-13	136
Tiefeng 31	Xin3511 × Resnick(from USA)	133
Tiefeng 33	Tie89059-8 × Xin3511	131
Tiefeng 35	Tie91017-6 × Jin8412	134
Tiedou 36	Tie90009-4 × Tie89078-10	130
Tiefeng 37	Tie91017-6 × Jin8412	134
Kaiyu 11	Kaijiao7528-36-4 × Kanzhimi	120–125
Kaiyu 12	–	110
Kaiyu 13	Xin3511 × K10-93	125
Kaijiao 8,157	Kaixi8525-26 × Kaijiao8157-3-3-1	125–130
Ji 1,005	Tongnong73-149 × Dandou5	135
Liaodou 21	Liao8878 × Liao93009	128
Liaodou 15	Liao85062 × Zhengzhouchangye-18	133
Liaoxian 1	–	105–110
Xinfeng 1	Changnong4 × Qunxuan1	121
Suke 1	–	136
Beijiang 1	Beihudou × Beifeng3	85–90
Hefeng 40	Beifeng9 × Hefeng34	113
Heinong 48	Ha90-6,719 × Sui90-5,888	118

And in the field-culture experiment, 25 soybean cultivars were further tested. The soaked seeds were sowed into soil ridges in the field, arranged in a randomized complete block design with six replicates^49^. The farm management was also similar to that of the pot-culture experiment.

### Sampling and chemical analysis

Plants were firstly washed thoroughly three times with tap water to remove soil and then carefully washed with de-ionized water for approximately 3 min. The roots, stems, leaves, bean-pods and seeds were separated. The samples were dried at 105 °C for 5 min, and then at 70 °C in an oven until completely dried. Every part biomass of plants were weighed and then were ground to power. Soil samples were air-dried and ground using a mortar and pestle, and then passed through a 0.149 mm sieve. The plant and soil samples were digested with a solution containing 87% of concentrated HNO_3_ and 13% of concentrated HClO_4_ (v/v)^52^. The concentration of Cd was determined by using an atomic absorption spectrophotometer (AAS, Hitachi 180-80 type, made in Japan). The detection limit for the heavy metal analysis is 0.01 mg kg^−1^. A certified reference material, soybean material (GBW10013, Qinghai Province, China) was used to monitor the recovery of metals from the plant samples. The geochemistry standard samples were used in this study to validate the soil analyses^33^. The recovery rates for the certified references, material soybean material and geochemistry standard sample, were 98% and 99%, respectively.

### Safety standard and statistical methods

According to the National Food Hygienic Standard of China (NFHSC), the maximum permissible concentration (MPC) of Cd (GB2715-2005) in grains for safe consumption is 0.2 mg kg^−1^ dry weight (DW).The standard was employed to evaluate the safety of consuming the seeds of the tested soybean cultivars grown in heavy metal contaminated soil.

For all crops, only the edible portions were sampled as the work was focused on the soil–plant–human pathway of trace metals. Thus, in this study, to evaluate the ability of a plant accumulating heavy metals Cd from soil to seed, the enrichment factor (EF)^35,53^ was calculated as follows:

$${\text{EF}} = {\text{ C}}_{{{\text{seed}}}} /{\text{ C}}_{{{\text{soil}}}}$$

where C_seed_ is the average Cd concentration (DW) of the seed of each cultivar, and C_soil_ is the total Cd concentration in corresponding soil.

To evaluate the transfer potential of Cd from root to seed, we calculated the translocation factors (TF)^40,41^ as follows:

$${\text{TF}} = {\text{ C}}_{{{\text{seed}}}} /{\text{C}}_{{{\text{root}}}}$$

where C_seed_ is the average Cd concentration (DW) of the seed of each cultivar, and C_root_ is the average Cd concentration (DW) of the root of each corresponding cultivar.

Data were analyzed by using the Excel 2003 and SPSS 13.0. All the values are expressed as mean ± standard deviation (S.D.) of the three replicates. Differences were considered significant at P < 0.05. Data were analyzed by one-way ANOVAS with the Duncan’s multiple range tests to separate means. All results were expressed on a dry weight basis.
